# Air pollution, health and regulations in Brazil: are we progressing?

**DOI:** 10.1590/0102-311XEN172924

**Published:** 2025-04-11

**Authors:** Rafael Junqueira Buralli, Patrick Connerton

**Affiliations:** 1 Faculdade de Saúde Pública, Universidade de São Paulo, São Paulo, Brasil.

Air pollution kills thousands of Brazilians every year, a number that increases as the years go by ^1^. The levels of pollutants observed in a large part of the country are alarming, as a result of the unhealthy combination of industrial contaminants, a large and old vehicle fleet, and the wildfires ^2^ that have always existed - which are not normal - but are increasing recently ^3^.

Air pollution is estimated to cause 8 million deaths every year globally, being the leading environmental risk factor for death. Annually, air pollution is responsible for more than 700,000 deaths among children under five years old - about 2,000 child deaths every day ^4^. But the health consequences go far beyond deaths and include several acute and chronic diseases, such as cardiovascular diseases, respiratory diseases, heart attacks, diabetes, cognitive changes, dementia and cancer ^4^ ^,^ ^5^. Pollution impacts people’s health and quality of life, healthcare services, the economy and productivity, generating costs of about USD 8.1 trillion in 2019, that is, 6.1% of the global gross domestic product (GDP) ^6^.

In Brazil, air pollution is a serious public health problem in urban and rural areas, causing about 326,478 deaths between 2019 and 2021 ^7^. Evidence indicates the relation of exposure to air pollution with hospitalizations and deaths in cities, especially among older adults ^8^. Seasonal fires are currently the main source of air pollutant emissions in central Brazil and the Amazon, in which about 60% of residents are exposed to unsafe air pollution levels for six months of the year ^3^, particularly affecting children and older adults ^3^ ^,^ ^9^. The most vulnerable and poorer populations ^10^ suffer from health impacts of air pollution the most, combining social and environmental injustice, aggravated by climate change.

One may struggle to assimilate how children living in the Amazon, which is considered by many as “the lungs of the planet”, can breathe, during many months every year, air with levels of pollution that could cause schools to cancel classes for a few days in California (United States) ^11^. But in Brazil, schools are not always safe from pollution. A study estimated exposure to pollutant sources in 186,080 Brazilian schools and identified that 25% of them are located less than 250m from main roads and/or have ≥ 7 records of wildfires within a radius of 10km, possibly exposing about 10 million students to very high pollution levels ^12^.

As long as necessary structural changes do not happen ₋ such as reducing fires and other sources of pollution, better resources to evaluate and ensure safe air conditions in schools using physical barriers or filtration systems - life goes on with little to no attention and mobilization concerning the issue, even with the certainty of negative impacts on the health and quality of life of the population. These pollutants are not restricted to the geographical limits of where they are released, but they travel long distances around the world ^13^. Thus, wildfires in the Amazon or Cerrado can impact the health of someone living far away, in addition to the local population ^2^.

Addressing the health effects of air pollution requires bold and articulated policies and actions on international, national, and local management levels. In 2021, the World Health Organization (WHO) updated its *Global Air Quality Guidelines* with ambitious targets that significantly reduce previous limits and consider the robust body of evidence demonstrating health effects even at very low pollution levels ^14^ ^,^ ^15^.

In this regard, Brazil has recently taken important steps in regulating air pollution, including *Law n. 14,850/2024* ^16^, which instituted the Brazilian National Air Quality Policy; and the new *Resolution n. 506/2024* ^17^, published by the Brazilian National Council for the Environment (CONAMA, acronym in Portuguese), which defined air quality standards and guidelines for enforcement. In 2022, the Supreme Court considered the current standards as “*insufficient for the rights to information, health and an ecologically balanced environment*” and determined that Brazil should review its goals by 2024 ^18^. Close to missing the deadline, the aforementioned resolution was presented by the Brazilian Ministry of the Environment as a “*result of 30 years of debates*” and a great advance because “*every year that we postpone deadlines and lawsuits means more deaths and a loss of life expectancy*” ^18^. Nevertheless, the proposed deadlines have four Intermediate Standards (IS), which are very loose and permissive, with consecutive reductions before the Final Standard, which is aligned with WHO recommendations. It would be reasonable if not for the long deadlines. As it stands, the last IS will only come into force in 2044 and there is established date to adopt WHO limits since the deadline for implementation of the Final Standard should be defined later in a “*date to be determined by CONAMA!!*” ^17^.

Despite the enthusiasm for the new policy, Brazil should have been more ambitious to be at the global forefront ^19^ and adequately protect population’s health. About 8,409 deaths could be avoided per year in the city of São Paulo alone if the limits recommended by WHO were met ^10^. From 2000 to 2019, Brazil lost more than 9 million productivity-adjusted life years and USD 268.05 billion due to premature deaths caused by exposure to the fine particulate matter (PM_2.5_); more than half of these could be avoided if WHO limits were met ^20^.

But there are still other layers of complexity in this public health issue. Brazil does not have the capacity to adequately monitor the situation, as it has a very limited air quality monitoring network restricted to a few capitals and large cities. Although these monitoring networks have been prescribed since 1989 by the CONAMA *Resolution n. 5/1989* ^21^, there is a data gap on air quality measured using official monitoring stations in Brazil, especially in the Central-West, North and Northeast. Notably, some states do not have an official single monitoring station recognized as adequate by legislation ^22^.

Properly monitoring air pollution and health effects is essential to promote environmental justice and reduce the burden of disease as it helps to reduce pollutant levels, especially when associated with public policies and popular mobilization actions ^23^. However, the lack of data on air quality in Brazil renders the problem invisible - almost intentionally. Thus, new technologies, like low-cost sensors and satellite data, have boosted the monitoring of pollutants and the analysis of health effects ^24^. These systems provide estimates that, associated with more robust official monitoring stations, help increase monitoring coverage and resolution.

Other important measures include carrying out and disseminating local emissions inventories by sector; promoting more sustainable and efficient industrial and transportation processes ^2^; improving urban and transportation planning; ensuring equitable access to green areas and resilience to climate change ^10^ ^,^ ^24^. In critical pollution episodes, health communication and prevention measures should be intensified, like avoiding outdoor physical activities, wearing masks and using air purifiers. It is essential to empower people and ensure social participation, improve communication on the effects of air pollution, and promote mitigation and control strategies. Recent reports, infographics and the Brazilian Ministry of Health monitoring panel deserve to be highlighted. Note that, it is necessary to define the potential problems related to pollution, use notification strategies (where, how and when to register) and qualify health workers to identify critical events and guide the population on how to protect themselves.

However, the current scenario of pollutant emissions raises doubts about the ability of many regions to reach the loose Brazilian standards. During the critical wildfire season, several regions of the country far exceeded the proposed initial limit ^25^. In addition, in cases in which limits are not respected, legislation does not prescribe what the consequences would be for states and municipalities that do not seek to meet the current standards. It is difficult to be optimistic when many states have not even published their Emission Control Plans, required since 2018 by the CONAMA *Resolution n. 491/2018* ^26^.

Furthermore, with the new CONAMA 2024 air pollution standards, Brazil ignored WHO recommendations, postponed the implementation of truly safe limits and froze its standards for 20 years, sending the message that the issue holds neither importance nor urgency, which is more difficult to believe when the sky is covered in toxic smoke from wildfires. CONAMA updated air pollution targets are not justified in terms of human health and do not protect the population, especially the most vulnerable. It may be too late to wait until 2044 to breathe safe air.
