# Supplementary material for: Air pollution, health and regulations in Brazil: are we progressing?
Source: Cad Saude Publica. 2025 Apr 11;41(3):e00172924. doi: 10.1590/0102-311XEN172924 (PMC11996184; doi:10.1590/0102-311XEN172924)
Supplement: Translation

# Translation: Poluição do ar, saúde e regulação no Brasil: estamos avançando?

**DOI:** 10.1590/0102-311XPT172924

**Authors:** Rafael Junqueira Buralli, Patrick Connerton

**Affiliations:** 1 Faculdade de Saúde Pública, Universidade de São Paulo, São Paulo, Brasil.

A poluição do ar mata milhares de brasileiros todos os anos ^1^. São alarmantes os níveis de poluentes observados em grande parte do país, resultados da combinação insalubre de contaminantes industriais, de frota veicular numerosa e antiga, e das queimadas ^2^, que sempre existiram - o que não é normal -, mas que estão aumentando muito recentemente ^3^.

Estima-se que a poluição do ar cause 8 milhões de mortes todos os anos globalmente, sendo o principal fator de risco ambiental de óbito. Anualmente, a poluição do ar é responsável por mais de 700 mil mortes entre crianças com menos de cinco anos - cerca de 2 mil mortes de crianças todos os dias ^4^. No entanto, as consequências na saúde vão muito além dos óbitos e incluem diversas doenças agudas e crônicas, como doenças cardiovasculares, respiratórias, infarto, diabetes, alterações cognitivas, demência e câncer ^4^ ^,^ ^5^. A poluição impacta a saúde e a qualidade de vida das pessoas, os serviços de saúde, a economia e a produtividade, gerando custos econômicos de cerca de USD 8,1 trilhões em 2019, ou seja, 6,1% do produto interno bruto (PIB) global ^6^.

No Brasil, a poluição do ar é um grave problema de saúde pública nas cidades e no campo, com cerca de 326.478 óbitos causados só entre 2019 e 2021 ^7^. Evidencias apontam a relação da exposição à poluição do ar com hospitalizações e óbitos em cidades, especialmente entre idosos ^8^. Queimadas sazonais são atualmente a principal fonte de emissão de poluentes atmosféricos na região central do Brasil e na Amazônia, onde cerca de 60% dos moradores estão expostos a níveis inseguros de qualidade do ar por seis meses no ano ^3^, afetando particularmente crianças e idosos ^3^ ^,^ ^9^. E os impactos da poluição do ar na saúde são maiores entre as populações mais vulneráveis e de menor renda ^10^, combinando injustiça social e ambiental, agravadas pelas mudanças climáticas.

Não é fácil entender como crianças que vivem na Amazônia, por muitos considerada o pulmão do mundo, podem respirar durante meses, todos os anos, a níveis de poluição que poderiam, por exemplo, fazer escolas cancelarem as aulas por alguns dias na Califórnia (Estados Unidos) ^11^. Mas no Brasil, nem sempre as escolas são um lugar seguro contra a poluição. Um estudo estimou a exposição a fontes de poluentes em 186.080 escolas brasileiras e identificou que 25% delas estão localizadas a menos de 250m de via principal de trânsito e/ou têm ≥ 7 registos de incêndios florestais num raio de 10km, expondo possivelmente cerca de 10 milhões de estudantes a níveis bastante elevados de poluição ^12^.

Enquanto mudanças estruturais necessárias não acontecem, como reduzir as queimadas e outras emissões, avaliar e garantir um ar seguro nas escolas por barreiras físicas ou sistemas de filtragem, a vida segue com pouco ou nenhum alarde e mobilização sobre o problema, mesmo com a certeza dos impactos na saúde e qualidade de vida da população. Esses poluentes não ficam restritos aos limites geográficos de onde são produzidos, mas transitam a longas distâncias e globalmente ^13^. Assim, as queimadas na Amazônia ou no Cerrado, além da população local, podem impactar a saúde de alguém vivendo bem longe dali ^2^.

Para enfrentar os efeitos da poluição do ar na saúde são necessárias políticas e ações ousadas e articuladas em todos os níveis de gestão, nas esferas internacional, nacional e local. Em 2021, a Organização Mundial da Saúde (OMS) atualizou suas *Diretrizes Globais de Qualidade do Ar* com metas ambiciosas que reduzem significativamente os limites anteriores e consideram o conjunto robusto de evidências que demonstram efeitos à saúde até em níveis de poluição muito baixos ^14^ ^,^ ^15^.

Nesse sentido, o Brasil recentemente deu passos importantes na regulação da poluição do ar, incluindo a *Lei nº 14.850/2024* ^16^, que instituiu a Política Nacional de Qualidade do Ar; e a nova *Resolução nº 506/2024* ^17^ do Conselho Nacional do Meio Ambiente (CONAMA), que definiu padrões de qualidade do ar e diretrizes para aplicação. Em 2022, o Supremo Tribunal Federal (STF) considerou os padrões vigentes “*insuficientes aos direitos à informação, à saúde e ao meio ambiente ecologicamente equilibrado*” e determinou que o Brasil revisasse suas metas até 2024 ^18^. Quase perto de esgotar o prazo, a referida Resolução foi apresentada pelo Ministério do Meio Ambiente e Mudança do Clima como “*resultado de 30 anos de debates*” e grande avanço pois “*cada ano que postergamos a entrada de prazos e ações significa mais mortes e perda de expectativa de vida*” ^18^. Não obstante, os prazos propostos têm quatro Padrões Intermediários (PI), muito elevados e permissivos, com reduções consecutivas antes do Padrão Final, este sim, alinhado com as recomendações da OMS. Seria até razoável se os prazos não fossem tão longos. Como está, o último PI só entrará em vigor em 2044 e não há previsão para adotar os limites da OMS, visto que o prazo de implementação do Padrão Final deverá ser definido posteriormente em “*data a ser determinada pelo CONAMA*”!! ^17^.

Apesar do entusiasmo com a nova política, o Brasil deveria ter sido mais ambicioso para estar na vanguarda global ^19^ e proteger adequadamente a saúde da população. Cerca de 8.409 mortes poderiam ser evitadas, por ano, só na cidade de São Paulo, se os limites recomendados pela OMS fossem cumpridos ^10^. De 2000 a 2019, o Brasil perdeu mais de 9 milhões de anos de vida ajustados pela produtividade e USD 268,05 bilhões devido à morte prematura por exposição ao material particulado fino (MP_2,5_); sendo que mais da metade poderia ser evitada se as orientações da OMS fossem atendidas ^20^.

Mas ainda há outras camadas de complexidade neste enorme problema de saúde pública. O Brasil não tem capacidade de monitorar adequadamente a situação, pois detém uma rede de monitoramento de qualidade do ar muito limitada, restrita a algumas capitais e grandes cidades. Apesar dessas redes de monitoramento serem previstas desde 1989 pela *Resolução nº 5/1989* ^21^ do CONAMA, há um vazio de dados sobre a qualidade do ar medida a partir de estações de monitoramento oficiais no Brasil, especialmente no Centro-oeste, Norte e Nordeste. Há estados que não têm sequer uma estação de monitoramento oficial que a legislação reconheça como adequada ^22^.

Monitorar adequadamente a poluição do ar e os efeitos à saúde é essencial para promover a justiça ambiental e reduzir a carga das doenças, pois ajuda a reduzir os níveis de poluentes, especialmente quando associado a políticas públicas e ações de mobilização popular ^23^. Mas, no Brasil, a falta de dados sobre a qualidade do ar invisibiliza - quase intencionalmente - o problema. Assim, as novas tecnologias têm impulsionado o monitoramento dos poluentes e as análises dos efeitos sobre a saúde, como os sensores de baixo custo e dados de satélite ^24^. Esses sistemas fornecem estimativas que, em conjunto com estações de monitoramento oficiais mais robustas, ajudam a aumentar a cobertura e a resolução do monitoramento.

Outras medidas importantes incluem realizar e divulgar inventários locais de emissões, por setor; promover processos industriais e de transporte mais sustentáveis e eficientes ^2^; melhorar o planejamento urbano e de transporte; garantir acesso equitativo a áreas verdes e resiliência às mudanças climáticas ^10^ ^,^ ^24^. Nos episódios críticos de poluição, devem ser intensificadas a comunicação em saúde e medidas de prevenção, como evitar atividade física em locais abertos, usar máscaras e filtros de purificação. É essencial empoderar as pessoas e garantir a participação social, melhorar a comunicação sobre os efeitos da poluição do ar e promover estratégias de mitigação e controle. Os recentes informes, os infográficos e o painel de monitoramento do Ministério da Saúde merecem destaque. Mas é preciso definir os potenciais agravantes relacionados à poluição, estratégias de notificação (onde, como e quando registrar) e qualificar os trabalhadores da saúde para identificar eventos críticos e orientar a população sobre como se proteger.

Entretanto, o cenário atual de emissão de poluentes gera dúvidas sobre a capacidade de muitas regiões atingirem os flácidos padrões brasileiros. Durante a temporada crítica de queimadas, diversas regiões do país excedem muito o limite inicial proposto ^25^. Ademais, nos casos em que os limites não são respeitados, a legislação não deixa claro quais seriam as consequências para estados e municípios que não buscarem atender os padrões vigentes. É difícil ser otimista quando muitos estados sequer publicaram seus Planos de Controle de Emissões, exigidos desde 2018 pela *Resolução nº 491/2018* ^26^ do CONAMA.

Outrossim, com os novos padrões de poluição do ar do CONAMA (2024), o Brasil ignorou as recomendações da OMS, postergou a implementação de limites realmente seguros e engessou seus padrões por 20 anos, passando a mensagem de que o tema não é importante ou urgente, o que é mais difícil de acreditar quando o céu fica encoberto pela fumaça tóxica das queimadas. As metas de poluição do ar atualizadas do CONAMA não se justificam em termos de saúde humana e não protegem a população, especialmente os mais vulneráveis. Pode ser tarde demais respirar um ar seguro só a partir de 2044.
